# Nuclear genetic codes with a different meaning of the UAG and the UAA codon

**DOI:** 10.1186/s12915-017-0353-y

**Published:** 2017-02-13

**Authors:** Tomáš Pánek, David Žihala, Martin Sokol, Romain Derelle, Vladimír Klimeš, Miluše Hradilová, Eliška Zadrobílková, Edward Susko, Andrew J. Roger, Ivan Čepička, Marek Eliáš

**Affiliations:** 1 Department of Biology and Ecology, Faculty of Science, University of Ostrava, Chittussiho 10, 710 00 Ostrava, Czech Republic; 2 Unité d’Ecologie, Systématique et Evolution, Centre National de la Recherche Scientifique (CNRS), Université Paris-Sud/Paris-Saclay, AgroParisTech, Orsay, France; 3 Institute of Molecular Genetics, Academy of Sciences of the Czech Republic, Vídeňská 1083, 142 20 Prague, Czech Republic; 4 Department of Zoology, Faculty of Science, Charles University, Viničná 7, 128 00 Prague, Czech Republic; 5 Department of Mathematics and Statistics, Dalhousie University, Halifax, NS B3H 4R2 Canada; 6 Centre for Comparative Genomics and Evolutionary Bioinformatics, Dalhousie University, Halifax, NS Canada; 7 Department of Biochemistry and Molecular Biology, Dalhousie University, Halifax, NS B3H 4R2 Canada; 8 Canadian Institute for Advanced Research, Program in Integrated Microbial Biodiversity, Toronto, ON Canada

**Keywords:** Codon reassignment, Evolution, Evolutionary constraint, Fornicata, Genetic code, *Iotanema spirale*, *Lygus hesperus*, Protists, Rhizaria, Transcriptome

## Abstract

**Background:**

Departures from the standard genetic code in eukaryotic nuclear genomes are known for only a handful of lineages and only a few genetic code variants seem to exist outside the ciliates, the most creative group in this regard. Most frequent code modifications entail reassignment of the UAG and UAA codons, with evidence for at least 13 independent cases of a coordinated change in the meaning of both codons. However, no change affecting each of the two codons separately has been documented, suggesting the existence of underlying evolutionary or mechanistic constraints.

**Results:**

Here, we present the discovery of two new variants of the nuclear genetic code, in which UAG is translated as an amino acid while UAA is kept as a termination codon (along with UGA). The first variant occurs in an organism noticed in a (meta)transcriptome from the heteropteran *Lygus hesperus* and demonstrated to be a novel insect-dwelling member of Rhizaria (specifically Sainouroidea). This first documented case of a rhizarian with a non-canonical genetic code employs UAG to encode leucine and represents an unprecedented change among nuclear codon reassignments. The second code variant was found in the recently described anaerobic flagellate *Iotanema spirale* (Metamonada: Fornicata). Analyses of transcriptomic data revealed that *I. spirale* uses UAG to encode glutamine, similarly to the most common variant of a non-canonical code known from several unrelated eukaryotic groups, including hexamitin diplomonads (also a lineage of fornicates). However, in these organisms, UAA also encodes glutamine, whereas it is the primary termination codon in *I. spirale*. Along with phylogenetic evidence for distant relationship of *I. spirale* and hexamitins, this indicates two independent genetic code changes in fornicates.

**Conclusions:**

Our study documents, for the first time, that evolutionary changes of the meaning of UAG and UAA codons in nuclear genomes can be decoupled and that the interpretation of the two codons by the cytoplasmic translation apparatus is mechanistically separable. The latter conclusion has interesting implications for possibilities of genetic code engineering in eukaryotes. We also present a newly developed generally applicable phylogeny-informed method for inferring the meaning of reassigned codons.

**Electronic supplementary material:**

The online version of this article (doi:10.1186/s12915-017-0353-y) contains supplementary material, which is available to authorized users.

## Background

The original assumption that all organisms use the same (standard) genetic code to translate genes to proteins has been challenged by discoveries of code deviations in both prokaryotes and, particularly, eukaryotes [1–3]. However, the richness of different genetic code variants in eukaryotes concerns primarily the mitochondrial translation system, whereas non-standard codes employed to translate plastid and nuclear genes have proven to be much rarer and less diverse [3, 4]. Discovering non-canonical genetic codes of eukaryotic nuclear genomes has a somewhat discontinuous history. The list of identified variants expanded steadily since the discovery of the first case in the mid 1980s [5, 6] until the early 2000s [7], with ciliates being an especially rich source. The variants described in this first period could be classified into three main types: (1) simultaneous reassignment of both the UAA and UAG stop codons (i.e., UAR) to encode glutamine (in some ciliates, some ulvophytes, a subgroup of diplomonads, and some oxymonads [1, 8]) or glutamate (again in ciliates [7]); (2) reassignment of the UGA stop codon to encode tryptophan or cysteine in different ciliate lineages [9]; and (3) reassignment of the CUG codon to encode serine rather than leucine in a yeast clade typified by *Candida albicans* [10]. In the subsequent period until very recently, a few new occurrences of the previously discovered code variants were reported from additional taxa [11, 12], most notably the stop-to-glutamine reassignment of UAR found in *Amoeboaphelidium protococcarum*, an algal parasite representing the Fungi-related group Aphelidea [13]. However, despite the dramatic rise of the amount of sequence data from diverse eukaryotes during this period, discovery of new variants of the nuclear genetic code lagged for 13 years.

The situation has changed dramatically in 2016 with a publication of a cluster of papers that have increased the number of known nuclear genetic code variants by a factor of two. Firstly, two independent studies [14, 15] demonstrated that the yeast *Pachysolen tannophilus* uses CUG to encode alanine rather than leucine. Next, analyses of a wealth of new transcriptome sequence data from ciliates, also by two independent teams [16, 17], led to a discovery of additional code variants in this group. One has UAR reassigned to encode tyrosine, others are unprecedented in that all three termination codons are employed to encode amino acids, specifically glutamine (UAR) and tryptophan (UGA). In *Condylostoma magnum* (Heterotrichea), UAG, UAA, and UGA all have a context-dependent dual meaning, as they still signal translation termination when occurring at the end of a coding sequence [16, 17]. In an unrelated ciliate, *Parduczia* sp. (Karyorelictea), UAR codons seem to exclusively specify glutamine, whereas UGA is used dually as a tryptophan or a termination codon [16]. Most recently, our group has reported a similar phenomenon from a somewhat unexpected area of the eukaryote phylogeny, a clade of trypanosomatids currently assigned to the genus *Blastocrithidia* [18]. In these organisms, UAR codons have a context-dependent dual meaning as either glutamate or termination codons, whereas UGA seems to be exclusively used as a tryptophan codon.

In summary, explorations of eukaryote phylogenetic diversity have established several clear trends in the evolution of non-standard genetic codes in nuclear genomes. Firstly, the most frequent changes concern the UAR and UGA codons. The only known exceptions, i.e., changes in the meaning of a sense codon, are the reassignments of CUG from leucine to either serine or alanine, both of which occur in one broader group of yeasts and are thought to result from a unique underlying synapomorphy, a loss of a tRNA cognate for the CUG codon in an ancestor of the whole group [14]. Nevertheless, the apparent scarcity of genetic codes with reassignments of sense codons may reflect the fact that they are not immediately noticeable in sequence analyses.

Secondly, both UAR codons (UAG and UAA) always experience the same alteration of their meaning in nuclear genomes. Reading UAA as a sense codon while keeping UAG as a termination one copes with the problem of the UUA anticodon recognizing not only the UAA codon, but also UAG due to wobble pairing. Analogous discrimination of the AUA and AUG codons, encoding isoleucine and methionine, respectively, is possible only thanks to the presence of specifically modified nucleotides (differing between the main domains of life) at the first anticodon position of Ile-encoding tRNAs [19]. On the other hand, reading UAG while maintaining UAA as stop is more readily achieved, since the former codon can be specifically decoded by a tRNA with the anticodon CUA. This was experimentally demonstrated in *Tetrahymena*, where tRNA^Gln^(CUA) interacts with UAG but not with the UAA codon [20]. Indeed, exclusive reassignment of UAG as a sense codon has been documented in some mitochondria [21, 22], and certain prokaryotes employ UAG, but not UAA, to encode pyrrolysine [2]. However, the fact that the meaning of the UAG and UAA codons is the same in virtually all translation systems suggests the influence of additional constraints. These may relate to the critical role of release factors, i.e., proteins directly interacting with termination codons and liberating polypeptide chains from the final tRNA molecule. Archaeal and eukaryotic release factors (aRF1 and eRF1, respectively) recognize all three termination codons, whereas in eubacteria and eukaryotic organelles, RF1 reads UAG and UAA and RF2 reads UAA and UGA [23]. Stop-to-sense reassignment of a codon thus requires a loss of a release factor (a possibility relevant for eubacteria and organelles) or specific modifications of a release factor to disrupt its interaction with the reassigned codon(s).

Thirdly, the phylogenetic distribution of non-canonical nuclear genetic codes is extremely biased. For reasons that are still not completely clear, ciliates behave as a real evolutionary hot-spot for recurrent emergence of various code deviations, whereas most other eukaryotes are much more conservative in this regard. Swart et al. [16] systematically screened data from the Marine Microbial Transcriptome Sequencing Project (MMETSP), a collection of deeply-sequenced transcriptomes from several hundreds of phylogenetically diverse protists [24], but did not find any cases of a non-canonical genetic code outside ciliates. However, the recent discovery of a new code variant in trypanosomatids [18] suggests that the final census of non-canonical codes in eukaryotes has not yet been reached.

Here, we present a discovery of two variants of a new type of the nuclear genetic code, in which UAG is translated as an amino acid, while UAA is kept as a termination codon (along with UGA). Both codes were encountered in (putatively) endobiotic organisms – a hitherto unknown, uncultured rhizarian and a recently described member of Fornicata (Metamonada), the anaerobic flagellate *Iotanema spirale*. We discuss possible evolutionary scenarios for the origin of these novel code variants.

## Results and Discussion

### A new lineage of insect-dwelling Rhizaria

As reported in Záhonová et al. [18], the non-canonical code of *Blastocrithidia* spp. (Trypanosomatida) was discovered accidentally by one of us noticing that a transcriptome shotgun assembly (TSA) from the heteropteran *Lygus hesperus* [25] is contaminated by trypanosomatid sequences including in-frame termination codons. Surprisingly enough, looking for contigs in the TSA data from *L. hesperus* exhibiting in-frame termination codons revealed not only those with obvious trypanosomatid affinities, but also some that could not be readily assigned to any particular eukaryotic taxon when compared by blastx against the NCBI non-redundant protein sequence database. Therefore, we probed the TSA data from *L. hesperus* with a set of conserved proteins employed in previous phylogenomic studies of the global eukaryote phylogeny (see Methods for details) and detected 71 orthologs that clustered neither with metazoans nor with trypanosomatids in individual single-protein trees (Additional file 1: Table S1*A*-*C*). Most of them contained one or more in-frame UAG codons; 62 of these sequences branched with bootstrap support (BS) > 50% exclusively (in a clan sensu [26]) with homologs from Rhizaria, most often (46 sequences, 23 of which with BS at least 90%) as a sister group of the aggregative amoeba *Guttulinopsis vulgaris*. The remaining nine sequences clustered with homologs from other groups, but only in two cases such a grouping was supported by BS > 50%, and in both cases the non-rhizarian homologs were nested in a broader clan comprising primarily rhizarian sequences (Additional file 1: Table S1C). Despite inconclusive or contradicting phylogenetic evidence for rhizarian affinity of the nine sequences, they all likely come from the same organism as those with clear rhizarian affinity, as evidenced by the presence of at least one in-frame UAG codon in all of them. In addition, many of the genes in the reference set that we used for searching the TSA from *L. hesperus* (e.g., genes for ribosomal proteins) are highly expressed, so contamination of the TSA by more than one species would manifest by the presence of more than one ortholog of these genes. Putting aside the previously detected trypanosomatid contamination [18], no other eukaryotic orthologs were observed, as were no additional 18S rRNA sequences. Therefore, we assumed that the TSA from *L. hesperus* was contaminated by two different protist species – *Blastocrithidia* sp. (see [18]) and an unidentified rhizarian, “Rhizaria gen. sp. ex *Lygus hesperus*”, hereafter for simplicity referred to as the rhizarian exLh.

**Fig. 1:**
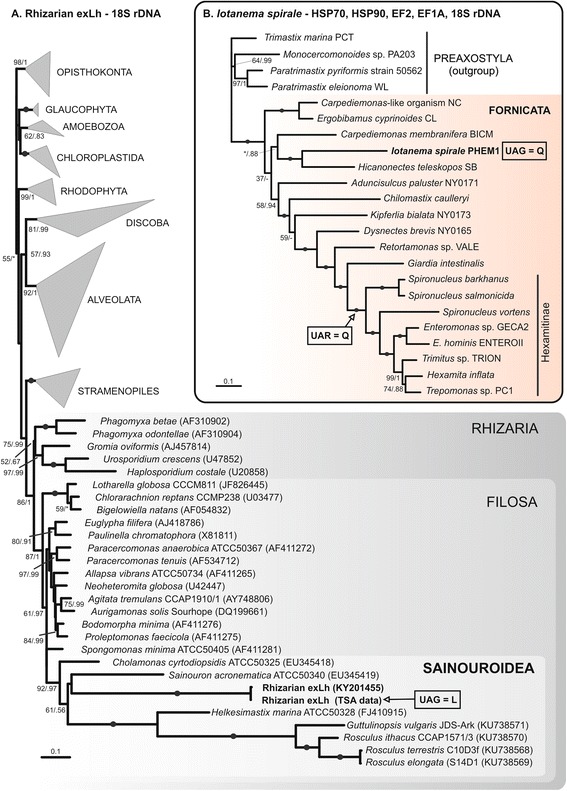
Phylogenetic position of the organisms studied. **a** Phylogeny of eukaryotes including the rhizarian exLh based on 18S rDNA sequences. The maximum likelihood (ML) tree was inferred with RAxML using the GTRGAMMAI substitution model. The values at branches represent RAxML BS values followed by PhyloBayes posterior probabilities (GTRCAT model). **b** Phylogeny of Fornicata including *I. spirale* based on a concatenated data set of 18S rDNA and EF-1α, EF2, HSP70, and HSP90 protein sequences. The ML tree was inferred with RAxML using the substitution models GTRGAMMA (for 18S rDNA) and PROTGAMMALG4X (for the protein sequences). The values at branches represent RAxML BS values followed by PhyloBayes posterior probabilities (CAT Poisson model). Maximal support (100/1) is indicated with black dots. Asterisks indicate support values lower than 50% or 0.5, respectively, dashes mark branches in the ML tree that are absent from the PhyloBayes tree

To further illuminate the identity of the rhizarian exLh, we carried out a maximum likelihood (ML) phylogenetic analysis of a 70-protein supermatrix containing 54 orthologs from this organism (a subset of the 71 genes mentioned above, passing an initially imposed threshold of minimal sequence identity to orthologs from other eukaryotes). The resulting tree showed it branching with maximum support within Rhizaria, specifically within Filosa as a sister lineage to *G. vulgaris* (Additional file 2: Figure S1). However, only very few representatives of Filosa could be included in the phylogenomic analysis due to an extremely low number of sequenced genomes or transcriptomes of this diverse group. Therefore, we sought to determine the phylogenetic position of the rhizarian exLh within Filosa using the 18S ribosomal RNA (rRNA) gene, the most broadly sampled phylogenetic marker for rhizarian phylogeny. Searching the *L. hesperus* TSA sequences revealed two contigs that proved to be chimeric sequences consisting from artificially merged parts of a different origin, including a 3’ segment of an 18S rRNA dissimilar to any 18S rRNA sequence in the GenBank database (and very different from the *L. hesperus* 18S rRNA sequence present in the TSA as another contig; see Methods for details). Using the partial 18S rRNA sequence as a seed and original RNA-seq reads we assembled a complete 18S rRNA sequence that fell phylogenetically into Filosa, specifically into the group Sainouroidea (Fig. 1a). This clade comprises several poorly studied free-living or coprophilic flagellates and amoebae, including *G. vulgaris* [27]. The result of the 18S rRNA analysis is thus concordant with the phylogenomic analysis of protein sequences, supporting the assumption that the assembled 18S rRNA sequence comes from the same organism as the protein-coding transcripts. Furthermore, it specifically indicates that the rhizarian exLh is a previously undetected lineage of Sainouroidea, presumably a separate genus.

The presence of sequences from a rhizarian species in the *L. hesperus* TSA may be explained by an accidental contamination or an error in data handling in the sequencing center. Alternatively, it may reflect a specific physical connection between *L. hesperus* and the rhizarian, most likely with the latter being a symbiont of the former. To distinguish between these possibilities, we obtained several individuals of *L. hesperus* captured in the wild (Kern County, CA, USA), isolated DNA from whole individuals, and carried out a PCR reaction using specific primers designed on the basis of the assembled 18S rRNA sequence of the rhizarian exLh. One of the reactions (with a template consisting of DNA pooled from five *L. hesperus* individuals) yielded a product of the expected size of 1200 bp, and sequencing revealed that, except for two substitutions, it was identical to the respective region of the 18S rRNA sequence obtained from the published transcriptomic data from *L. hesperus*. This result suggests that the rhizarian exLh is a natural inhabitant, perhaps an endobiont, of *L. hesperus*. The nature of this association (mutualism, commensalism, or parasitism) and the host range of the rhizarian exLh remain to be investigated.

### The rhizarian exLh employs UAG to encode leucine

**Fig. 2:**
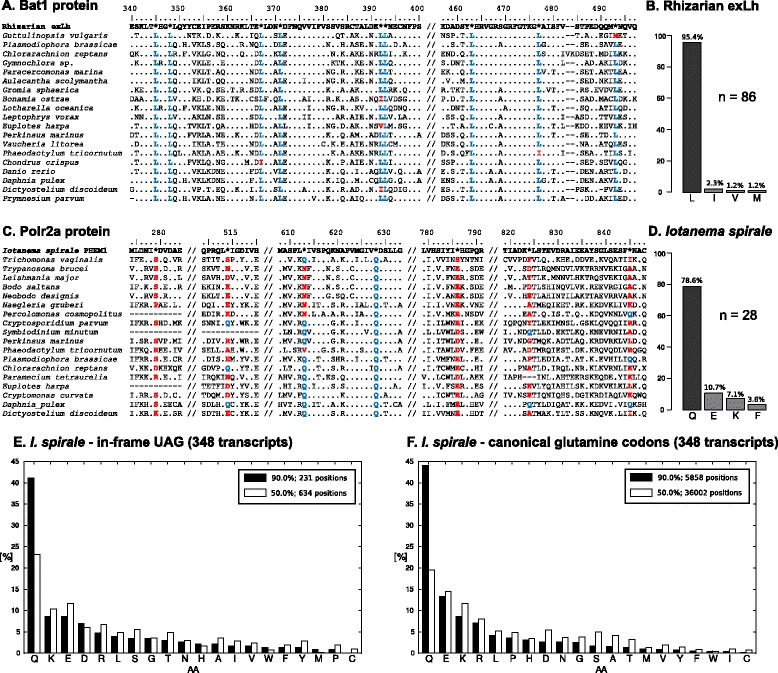
In-frame UAG codons in protein-coding genes of the rhizarian exLh and *I. spirale*. **a** An example of a rhizarian exLh gene with several in-frame UAG codons: multiple sequence alignment of orthologs of the Bat1 protein (spliceosome RNA helicase). **b** Relative frequency of hyperconserved positions (at least 90% amino acid identity across orthologs from 250 representatives of main eukaryotic groups in the alignment) corresponding to UAG-containing sites in the rhizarian exLh transcripts. **c** An example of a *I. spirale* gene with several in-frame UAG codons: multiple sequence alignment of orthologs of the Polr2a protein (also known as RNA polymerase II subunit RPB1). **d** Relative frequency of hyperconserved positions (at least 90% amino acid identity across orthologs from 54 representatives of main eukaryotic groups in the alignment) corresponding to UAG-containing sites in the rhizarian exLh transcripts. **e**, **f** Dominant amino acid identity at conserved alignment positions (defined using 90% and 50% threshold) in a broad-scale comparison of *I. spirale* sequences with eukaryotic homologs. **e** Positions corresponding to in-frame UAG codons in *I. spirale* sequences. **f** Positions corresponding to canonical glutamine codons (CAG, CAA) in *I. spirale* sequences. In Fig. 2**a** and **c**, only selected segments of the full alignments (separated by double slashes) are shown for simplicity. Asterisks indicate positions with in-frame UAG codons in the underlying coding sequences. In Fig. 2**b** and **d**, the hyperconserved positions are sorted according to the respective hyperconserved amino acid residue (only four most frequent position classes are shown). Source tables for Fig. 2**b** and **d** including data from read mapping are available in Additional file 1: Table S1D and S2C

We next investigated the identity and significance of the termination codons interrupting the coding sequences of the rhizarian exLh. In total, we identified 384 instances of in-frame termination codons in 71 genes (transcripts); the codon was UAG in all cases. Based on a comparison with orthologs from other eukaryotes, we identified the *bona fide* termination codons of the respective transcripts (i.e., the 3’-ends of the respective coding sequences), which were exclusively represented by UAA (56 cases) or UGA (15 cases) (Additional file 1: Table S1A–C). This suggested that the UAG codon has been reassigned as a sense codon in the rhizarian exLh and does not signal translation termination anymore. Visual inspection of multiple protein sequence alignments revealed a conspicuous pattern in the distribution of in-frame UAG codons – a strong tendency to occur at positions occupied by conserved leucine residues (for an example see the alignment of Bat1 protein sequences, Fig. 2a). We hypothesized that UAG encodes leucine in the novel rhizarian, which was further formally tested by two approaches.

For the first analysis, we inspected the alignments of orthologous protein sequences used for the phylogenomic analysis described above and identified 192 positions in well conserved blocks that corresponded to an in-frame UAG in the sequences from the rhizarian exLh. From those positions, 86 were classified as hyperconserved, i.e., with at least 90% amino acid identity across all the sequences in the alignment; 95% (i.e., 82) of which corresponded to a hyperconserved leucine (Fig. 2b). To ensure that the presence of the in-frame UAG codons at these hyperconserved sites was supported by original sequencing data, we inspected raw RNA-seq reads mapped onto the respective contigs. No conflicting signal concerning the identity of the nucleotides corresponding to any of these UAG codons was apparent (each in-frame UAG was supported by more than one read, with the read variability lower than 4.50%) (Additional file 1: Table S1D). As an alternative to the hyperconserved position-based inference of the UAG codon meaning, we devised a phylogeny-informed ML-based method that unselectively considers all UAG positions (see Methods for details). Briefly, we first inferred an organismal phylogeny using a smaller dataset of eight conserved proteins (to save computation time), with the respective genes from the rhizarian exLh containing 71 in-frame UAG codons, represented as an undetermined amino acid (X) in the alignment. We then prepared 20 modifications of the dataset, each with a different amino acid considered at positions corresponding to the in-frame UAG codons in the genes from the rhizarian exLh. Then, we calculated the best ML tree for each of the 20 datasets, using the same substitution model and the tree from the initial dataset as a constraint. The dataset where UAG was translated as leucine showed the highest likelihood score, and the conditional probability that UAG encodes leucine in the rhizarian exLh (calculated conditional upon UAG encoding one amino acid at all positions) is virtually 1.0, whereas it is negligible for any other amino acid (Table 1).

**Table 1:** Phylogeny-informed maximum likelihood-based estimation of the UAG codon meaning in the rhizarian exLh and *Iotanema spirale*. See the main text for details on the procedure employed. Note that the values of conditional probabilities for the most likely assignments (UAG = L in the rhizarian exLh and UAG = Q in *I. spirale*) differ so little from 1.00 that they must have been indicated as 1 subtracted by the sum of conditional probabilities of the 19 other assignments

Rhizarian exLh			*I. spirale*		
Alternative UAG meaning	Log-likelihood score	Conditional probability	Alternative UAG meaning	Log-likelihood score	Conditional probability
L	–362314.715	1 – (2.65 × 10^–82^)	Q	–55505.075	1 – (2.36 × 10^–34^)
I	–362502.554	2.65 × 10^–82^	E	–55582.654	2.03 × 10^–34^
V	–362543.823	3.16 × 10^–100^	K	–55584.47	3.31 × 10^–35^
M	–362553.384	2.23 × 10^–104^	R	–55632.34	5.36 × 10^–56^
F	–362585.699	2.06 × 10^–118^	S	–55657.799	4.71 × 10^–67^
A	–362632.167	1.4 × 10^–138^	A	–55659.005	1.41 × 10^–67^
T	–362657.807	9.9 × 10^–150^	N	–55672.232	2.54 × 10^–73^
Q	–362673.9	1.02 × 10^–156^	T	–55677.733	1.04 × 10^–75^
R	–362687.179	1.74 × 10^–162^	D	–55688.053	3.42 × 10^–80^
S	–362701.797	7.81 × 10^–169^	H	–55708.978	2.79 × 10^–89^
K	–362710.573	1.2 × 10^–172^	L	–55710.215	8.11 × 10^–90^
Y	–362713.231	8.45 × 10^–174^	P	–55750.281	3.22 × 10^–107^
P	–362724.093	1.62 × 10^–178^	G	–55753.721	1.03 × 10^–108^
H	–362741.154	6.31 × 10^–186^	V	–55757.545	2.26 × 10^–110^
C	–362745.196	1.11 × 10^–187^	M	–55785.355	1.89 × 10^–122^
E	–362745.729	6.50 × 10^–188^	I	–55808.382	1.89 × 10^–132^
W	–362766.438	6.59 × 10^–197^	Y	–55825.982	4.28 × 10^–140^
N	–362774.796	1.55 × 10^–200^	F	–55883.499	4.49 × 10^–165^
G	–362797.379	2.41 × 10^–210^	C	–55936.549	4.10 × 10^–188^
D	–362848.162	2.12 × 10^–232^	W	–55966.189	5.50 × 10^–201^

**Fig. 3:**
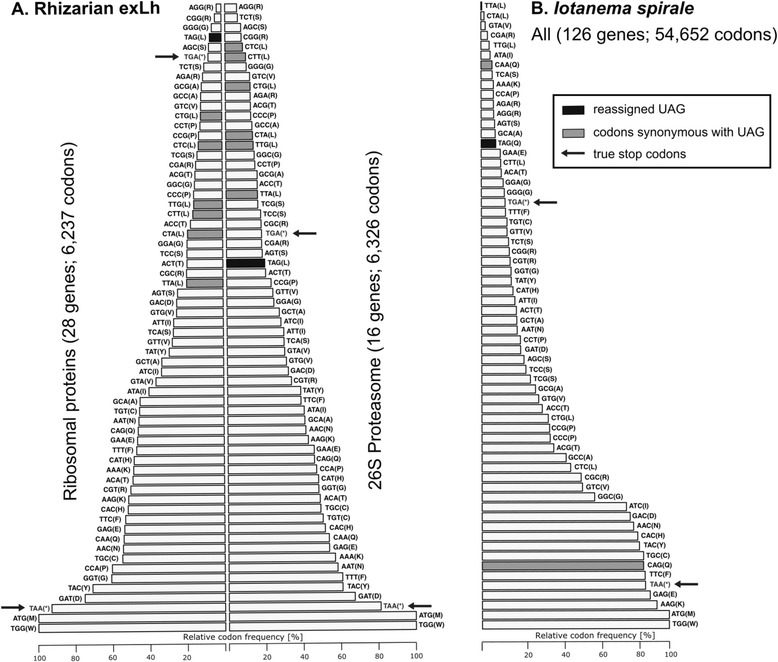
Relative codon frequencies in the rhizarian exLh and *I. spirale*. **a** Relative codon frequencies in two different groups of genes (for ribosomal proteins and for subunits of the 26S proteasome; listed in Additional file 1: Tables S1A and S1B) in the rhizarian exLh. **b** Relative codon frequencies in a reference set of genes of *I. spirale* (listed in Additional file 3: Tables S2A and S2B). The relative codon frequencies are calculated as the percentage of the codon among all occurrences of codons with the same meaning (i.e., coding for the same amino acid or terminating translation)

Based on these results, we conclude that UAG is the seventh codon for leucine in the rhizarian exLh and does not serve as a stop codon in this organism. This is not without precedent, as the stop-to-leucine UAG reassignment was previously reported from mitochondrial genomes of several green algae of the order Sphaeropleales [22, 28] and of the chytrid fungus *Spizellomyces punctatus* and its relatives [21]. Interestingly, we noticed striking differences in the UAG codon abundance between certain groups of genes from the rhizarian exLh. In-frame UAG codons were overrepresented in genes encoding components of the 26S proteasome, where the UAG codon was the most abundant codon for leucine (Fig. 3a). In contrast, UAG was the rarest leucine codon in genes for ribosomal proteins. In total, we identified sequences corresponding to 28 ribosomal protein genes of the rhizarian exLh, but 50% of those sequences did not contain any UAG codon, although they all branched with homologs from Rhizaria, most often (11/14) sisters to those from *G. vulgaris*. Genes for ribosomal proteins are highly expressed and typically exhibit a strong codon usage bias facilitating efficient synthesis of ribosomal proteins [29]. The low abundance of the UAG codon in ribosomal protein genes in the rhizarian exLh thus suggests that this codon is not as efficiently translated as the six standard codons for leucine.

### *I. spirale* (Fornicata) uses UAG to encode glutamine

The second case of a novel non-canonical genetic code was unexpectedly encountered when we sequenced the transcriptome of *I. spirale*. It is a recently described anaerobic flagellate isolated from fresh feces of a gecko *Phelsuma grandis* and is considered an intestinal endobiont [30]. Phylogenetic analyses based on a fragment (~1000 bp) of the 18S rRNA gene sequence affiliated *I. spirale* to the previously defined “*Carpediemonas*-like lineage 3” (CL3; [31]) in Fornicata (one of the three principal groups of Metamonada), although its morphology is highly unusual for a fornicate [30]. To corroborate this initial insight, we used the data from the newly sequenced transcriptome of *I. spirale* and carried out two multi-locus phylogenetic analyses. A 70-protein phylogenomic analysis (the same as used above for establishing the phylogenetic position of the rhizarian exLh) with the dataset including 60 orthologs from *I. spirale* showed this organism as a branch sister to, yet deeply diverged from, representatives of Diplomonadida (Additional file 2: Figure S1). This is consistent with the previous result based on the partial 18S rRNA gene sequence and with the fact that no other non-diplomonad fornicate could be included in the analysis due to lack of genome-scale sequence data. The second analysis was based on a dataset comprising a complete 18S rRNA sequence, which we identified as one of the assembled transcript contigs, and sequences of four conserved proteins used in a previous detailed study of the phylogeny of the Fornicata [32]. This analysis placed *I. spirale* with maximum support as the sister lineage of the CL3 representative *Hicanonectes teleskopos* (Fig. 1b), again congruently with the previously reported result. Thus, it is now robustly established that *I. spirale* is an unusual fornicate. In addition, it is a lineage well separated from Hexamitinae, a subgroup of diplomonads, which is a conclusion important for the interpretation of the evolution of the genetic code in fornicates (see below).

While analyzing the assembled transcript sequences from *I. spirale*, we noticed occasional in-frame UAG codons (for an example, see the alignment of Polr2a protein sequences, Fig. 2c). In total, we scrutinized sequences representing 126 genes, which contained 204 in-frame UAG codons (Additional file 3: Tables S2A and S2B). UAG was not used as an apparent *bona fide* termination codon in any of these transcripts, as 3’-termini of coding sequences predicted on the basis of conservation with orthologous sequences were marked only with UGA (in 15 cases) and, significantly, UAA (in 104 cases) (the remaining seven transcripts were truncated). This suggested that UAG, but not UAA, has been reassigned as an amino acid-encoding codon in *I. spirale*. We used similar approaches as employed for analyzing the genetic code of the rhizarian exLh to determine the identity of this UAG-encoded amino acid (see Methods for details). First, using a smaller subset of genes sampled broadly to include representatives from most major eukaryotic lineages we identified 28 hyperconserved positions with an in-frame UAG in *I. spirale* (in all cases confirmed by inspection of raw reads mapped onto the respective transcript), 22 of which (i.e., 79%) corresponded to a hyperconserved glutamine (Fig. 2a and Additional file 3: Table S2C). In the second analysis, a concatenated protein sequence alignment considering glutamine in place of in-frame UAG codons in *I. spirale* sequences gave the highest likelihood among all 20 possible variants (considering 20 different amino acids) when tested against a precomputed species tree, and the conditional probability that UAG encodes glutamine in *I. spirale* was nearly 1.0, with negligible probabilities obtained for any other amino acid (Table 1).

To further test that UAG is the only termination codon reassigned in *I. spirale* and that its meaning is to encode glutamine, we carried out a broader analysis of the available transcriptomic data (see Methods for details). In total, we analyzed 730 contigs assigned to *I. spirale* (i.e., apparently not coming from the prokaryotes contaminating the culture) and exhibiting close homologs in other eukaryotes; 348 of them contained at least one UAG codon within the region aligned to the homologs. Considering positions with 90% amino acid identity across the alignment with 50 best blastp hits, we identified 231 positions that corresponded to an UAG codon in the *I. spirale* sequence, 95 (41.13%) of which were dominated by glutamine (and no other amino acid reached such a frequency at the positions; Fig. 2e). Although this value may seem low and ambiguous, a similar proportion of conserved alignment positions dominated by glutamine (44.08%) was observed for positions occupied by canonical glutamine codons in *I. spirale* sequences (Fig. 2f). Imposing a less stringent threshold for amino acid conservation across the alignment (50%) yielded 634 positions with an UAG codon in the *I. spirale* sequence, 147 (23.19%) of which were dominated by glutamine. For canonical glutamine codons in *I. spirale* sequences the proportion was even lower (19.52%). In addition, neither of the 730 examined transcripts included the UAG codon as an obvious termination codon marking the end of the coding sequence.

All these results indicate that UAG in *I. spirale* encodes glutamine and does not serve as a stop codon. In contrast, our procedure identified only two contigs (a2783c02 and a9294c03) with candidate in-frame UAA or UGA codons, but manual scrutiny revealed that these codons are located in regions representing obvious retained introns. Thus, there is no evidence for reassignment or context-dependent dual meaning of the UAA or UGA codons in *I. spirale* and both apparently serve solely as standard termination codons, with UAA being the predominant termination codon in *I. spirale* (Fig. 3b). In the past, such a genetic code variant (UAG = Q, UAA = stop) was attributed to the ciliate genus *Blepharisma* by some sources, see, e.g., the code 15 (“Blepharisma Macronuclear”) in the list of genetic codes at an ftp page of NCBI [33] or a recent textbook on molecular and genome evolution [34]. However, this was an apparent error, as it is beyond any doubt that *Blepharisma* spp. use both UAA and UAG as stop codons, while they have reassigned UGA as a tryptophan codon [9, 16]. Indeed, the most recent list of genetic code tables provided on another NCBI page [35] omits the code 15. Thus, the code we here document for *I. spirale* is, to the best of our knowledge, truly unprecedented.

### Evolutionary origin of the non-standard genetic codes in the rhizarian exLh and *I. spirale*

Rhizaria is an extremely diverse eukaryotic grouping, but our knowledge of even the general biology, let alone molecular details, of most of rhizarian groups is lamentable. The discovery of a new rhizarian lineage exhibiting a peculiar feature of their gene expression machinery is thus not so unexpected. Our phylogenetic analyses place the new rhizarian with the stop-to-leucine reassignment of the UAG codon into the group Sainouroidea (see above). We should, therefore, ask how widespread this feature is in Sainouroidea or possibly a broader rhizarian clade. The closest relative of the rhizarian exLh, for which a substantial amount of sequences of protein coding genes (transcripts) are available, is *G. vulgaris*. Brown et al. [36] sequenced its transcriptome using the 454 method, but did not mention any observation concerning the genetic code employed by this organism. Therefore, we analyzed the 147 transcript sequences from *G. vulgaris* that have been released by Brown et al. [36] to the GenBank database (JT844885–JT845030). Only one of these sequences, JT844913, includes obvious in-frame termination codons (specifically a spot with UAG and UGA separated by a glutamine codon; see Additional file 4: Table S3), but checking unpublished Illumina RNA-Seq data from *G. vulgaris* revealed that the occurrence of the two termination codons in JT844913 is a sequencing error (Dr. M. Brown, personal communication). Interestingly, all but one available *G. vulgaris* transcript sequences that cover the 3’-end of the coding sequence use UAA as the termination codon, with the sole exception putatively employing UAG (Additional file 4: Table S3). Thus, *G. vulgaris* most likely does not share the stop-to-leucine UAG reassignment with the rhizarian exLh, although the prevalence of UAG as a termination codon needs to be investigated using more complete sequence data. Little data is available on nuclear protein-coding genes of other sainouroids, specifically a single sequence for each of *Rosculus* sp. (DQ388527.1), *Helkesimastix* (AY748812.1), and *Sainouron acronematica* (DQ098274.1). Neither of these sequences exhibits in-frame termination codons, and the gene from *Rosculus* sp. uses UAG as the *bona fide* termination codon, whereas the remaining two sequences have truncated 3’-ends. These sequences are, therefore, consistent with the notion that the genetic code has changed specifically in the rhizarian exLh lineage, but a systematic exploration of sainouroid transcriptomes or genomes is needed to pinpoint this evolutionary event with a higher confidence.

While our study uncovers the first case of a non-canonical nuclear code for the whole Rhizaria, the departure from the standard code reported here from *I. spirale* is not unprecedented in the Fornicata. Specifically, hexamitin diplomonads (Hexamitinae) for example, members of the genera *Spironucleus*, *Trimitus* or *Trepomonas*, also encode glutamine by non-standard codons [37, 38]. However, in contrast to *I. spirale*, all hexamitin taxa investigated have reassigned both UAG and UAA as glutamine codons. The non-hexamitin diplomonads of the genus *Giardia* are known to employ the standard genetic code, and our inspection of the limited number of protein-coding gene sequences available from the various “*Carpediemonas*-like” fornicates [32] did not reveal any case of an in-frame UAG (or UAA) codon that would presumably encode glutamine. Hexamitins and the *I. spirale* lineage thus apparently modified their genetic codes independently of each other (Fig. 1b). The closest *I. spirale* relative, from which sequences of protein-coding genes are available, is *H. teleskopos*. All four these sequences (GenBank accession numbers AB600290.1 to AB600293.1) lack in-frame UAG codons, suggesting that the stop-to-glutamine UAG reassignment occurred only after the *Iotanema* lineage split from the one of *Hicanonectes*. However, we need to be cautious, as the abundance of the UAG sense codon may be low (it corresponds to only ~8% of all glutamine codons in *I. spirale* genes; Fig. 3b), and we also lack positive evidence for UAG as a termination codon in *H. teleskopos* due to 3’-end truncation of all four sequences. It will be interesting not only to obtain more complete data for investigating the genetic code of *H. teleskopos*, but also to study the free-living unidentified isolate PCS [31] that is phylogenetically closer to *I. spirale* than *H. teleskopos* [30], and of members of the endobiotic genus *Caviomonas*, whose relationship to *I. spirale* has been suggested by morphological similarities of their flagellar apparati [30].

### Molecular mechanisms of codon reassignment in the rhizarian exLh and *I. spirale*

Let us now touch briefly upon the actual molecular underpinnings of the changed specificity of the UAG codon in the rhizarian exLh and *I. spirale*. The occurrence of UAG as a sense codon implies the existence of a cognate aminoacyl-tRNA that translates UAG into the proper amino acid. As this aminoacyl-tRNA must at the same time ignore UAA, which has been retained as a dominant stop codon in both taxa, we are left with a single possible anticodon, CUA, which pairs with the UAG codon but not with the UAA codon due to C:A mismatch at the first anticodon:third codon position. Therefore, we predict that sequencing the genomes of the rhizarian exLh and *I. spirale* will reveal the presence of novel tRNAs with the CUA anticodon and with attributes characteristic for tRNAs recognized by leucinyl-tRNA synthetase and glutaminyl-tRNA synthetase, respectively. Genes for tRNA^Leu^(CUA) were previously found in mitochondrial genomes of the chytrid *S. punctatus* [21] and several green algae of the order Sphaeropleales [22], so the postulated existence of a similar gene in the rhizarian exLh is not without precedent. Similarly, the occurrence of tRNA^Gln^(CUA) has already been documented, namely in ciliates with the stop-to-glutamine reassignment of UAR codons, e.g., *Tetrahymena thermophila* [38] or *C. magnum* [16], and in hexamitins, e.g., in the *Spironucleus salmonicida* genome (the tRNA gene SS50377_t0098 on the scaffold scf7180000020657; GenBank accession number KI546140.1). Notably, anticodons of these tRNAs differ in only one nucleotide position from anticodons of standard tRNAs carrying the respective amino acids, i.e., tRNA^Leu^(CAA) decoding the UUG leucine codon and tRNA^Gln^(CUG) decoding the CAG glutamine codon.

**Fig. 4:**
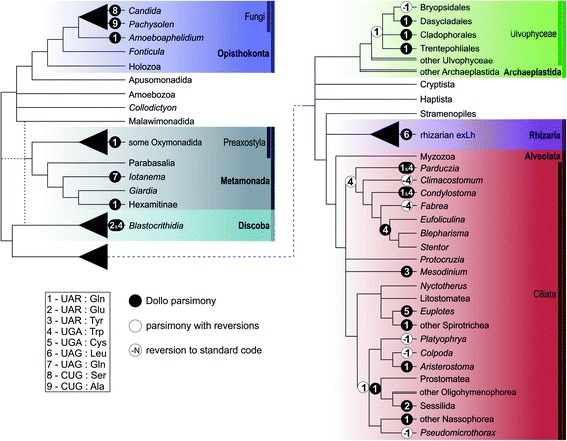
Phylogenetic distribution of known non-canonical genetic codes in nuclear genes of eukaryotes. The schematic phylogenetic tree was drawn on the basis of phylogenetic and phylogenomic analyses for eukaryotes as a whole [60, 71, 72] (our own Fig. 1 and Additional file 2: Figure S1) and for the relevant subgroups with non-canonical codes [12, 13, 73–77]. Multifurcations indicate uncertain or controversial branching order, dashed branches indicate different positions of Metamonada within eukaryotes suggested by different studies, branches drawn as double lines indicate paraphyletic groupings. The types and occurrences of the different non-canonical codes are based on this study (the rhizarian exLh and *Iotanema*) and the following previous reports: fungi [14, 15]; *Amoeboaphelidium* [13]; oxymonads [11]; *Blastocrithidia* [18]; ulvophytes [12]; ciliates [7, 9, 16, 17]. Note that, for simplicity, code variants with a context-dependent dual meaning of UAR or UGA codons as sense or termination ones (UAR in *Blastocrithidia* and *Condylostoma*, UGA in *Parduczia* and *Condylostoma*) are not distinguished from those with a “complete” reassignment. We also omitted some ciliate species with their putative non-canonical codes supported by little data that are specifically related to and possibly sharing the same code with better studied species. Changes in the genetic code are mapped onto the tree primarily (black circles) using Dollo parsimony (no reversions are allowed). An alternative maximum parsimony scenario with reversions weighted the same as other changes is indicated by the respective code numbers in white circles. An alternative branching order to the one indicated in the figure was supported by some studies for some of the ciliate lineages, but the alternative topology does not decrease the minimal number of codon reassignments required to explain the distribution of non-standard genetic codes

Hence, the simplest scenario for the evolutionary origin of the putative UAG-decoding tRNAs in the rhizarian exLh and *I. spirale* is a mutation of pre-existing (duplicated copies of) tRNA^Leu^(CAA) and tRNA^Gln^(CUG) genes, respectively. Checking a test set of 27 nuclear genomes confirmed that tRNA^Leu^(CAA) and tRNA^Gln^(CUG) commonly occur in eukaryotic genomes, often in multiple copies in the genome (Additional file 5: Table S4), which is conducive to the emergence of the postulated mutant variants required for reading the UAG codon. In addition, leucinyl-tRNA synthetases generally do not recognize the anticodon as a tRNA identity element [39–41], suggesting that efficient charging by leucine of the newly emerged tRNA^Leu^(CUA) does not necessarily require changes in the enzyme. In contrast, glutaminyl-tRNA synthetases do use the anticodon as a tRNA identity element, and indeed, tRNA^Gln^(CUA) from *Tetrahymena thermophila* was not recognized as a substrate by a mammalian glutaminyl-tRNA synthetase [42]. Unfortunately, to our knowledge, glutaminyl-tRNA synthetases from eukaryotes decoding UAG (and UAA) as glutamine have not been studied in detail, so the putative modifications needed for efficient charging of tRNA^Gln^(CUA) are unknown. Nevertheless, the multiple independent cases of the stop-to-glutamine UAG reassignment in various eukaryotes (Fig. 4) by themselves suggest that such modifications are achieved readily.

Identification of tRNAs cognate to the UAG codon in the rhizarian exLh and *I. spirale* and elucidation of their evolutionary origin awaits sequencing the genomes of the two organisms. This will require identification and culturing the rhizarian exLh; work towards this goal is underway in our laboratory. Sequencing the genome of *I. spirale* is complicated by the fact that it grows slowly and the culture is dominated by bacteria [30], which by itself rules out direct sequencing of tRNA molecules as an alternative approach. Moreover, it should be noted that identification of tRNAs responsible for reading reassigned termination codons may not be straightforward even when the genome sequence is available. For example, Swart et al. [16] failed to find a gene for the expected tRNA^Trp^(UCA) in their draft genome sequence of the ciliate *C. magnum* when investigating the genetic code of this organism. The specificity of tRNAs is not necessarily obvious from the gene sequence itself, as post-transcriptional editing or base modifications may be involved, too. As a result, the actual tRNAs responsible for termination codon reassignments remain unknown for most of the previously described non-canonical codes in eukaryotic nuclear genomes.

An effective reassignment of any of the UAG, UAA, or UGA codons is commonly thought to also depend on specific changes in the mechanism of translation termination. In eukaryotes, translation termination is mediated by the interaction of all three termination codons with the same protein, eRF1 (eukaryotic release factor 1), specifically with its N-terminal domain [43, 44]. This domain includes several highly conserved motifs or individual residues directly or indirectly involved in the recognition or binding of the termination codons, namely GTS, (TAS)NIKS, YxCxxxF, E55, and S70 [45, 46] (the residue numbering is based on the human eRF1 sequence as a reference). Indeed, eRF1 sequences in eukaryotes that have altered the meaning of UAG, UAA, or UGA codons proved to typically exhibit various alterations in these motifs when compared to eRF1 sequences from organisms with the canonical code [9, 47, 48], and some of these changes have been demonstrated as causally linked to an altered specificity of the eRF1 protein towards the termination codons [45, 49].

We identified transcripts encoding eRF1 in both the rhizarian exLh and *I. spirale* and compared the critical region of the respective eRF1 protein sequences with homologs from diverse eukaryotes, including a wide selection of species with non-canonical genetic codes (Additional file 6: Figure S2). The eRF1 sequence from the rhizarian exLh does not display any obvious deviation in the conserved elements noticed above, but it notably exhibits an alanine residue at the Leu69 position of the human eRF1 protein. Although this position is not particularly conserved among eRF1 proteins, the substitution to alanine is unique for the rhizarian exLh (Additional file 6: Figure S2) and a corresponding L69A mutation was shown to increase readthrough of all three stop codons, particularly of the UAG codon, suggesting that this position is specifically important for the recognition of guanine of the third stop codon position by eRF1 [45]. It is, therefore, possible that this substitution is partly responsible for efficient usage of UAG as a sense codon in the rhizarian exLh. The most conspicuous feature of the eRF1 protein from *I. spirale* is a mutation in the highly conserved GTS motif, specifically T32G substitution (Additional file 6: Figure S2). This seems to be significant. With adenosine in the second position of the termination codon, Thr32 faces the base at the third position, making a hydrogen bond with the N2 atom of guanosine in UAG [46]. Hence, the T32G substitution presumably disrupts this interaction and weakens the affinity of the *I. spirale* eRF1 to the UAG codon. The analysis of the eRF1 sequences from the rhizarian exLh and *I. spirale* thus provides interesting testable working hypotheses on how the translation apparatus of these two taxa has been modified to interpret the UAG codon as a sense one.

## Conclusions

Since the discovery of the first non-standard genetic code in ciliates more than 30 years ago until our study, all known nuclear genetic code alternations have followed a regular pattern that evolutionary changes in the meaning of the UAG and UAA codons are coupled. We mapped the distribution of documented non-standard genetic codes in eukaryotic nuclear genomes onto the species phylogeny and deduced the most parsimonious scenario explaining the origin of these codes (Fig. 4). The analysis predicts at least 13 independent evolutionary changes in the meaning of coordinately in both UAG and UAA (including possible reversions to the standard code or a putative change of an encoded amino acid from glutamine to glutamate in a particular ciliate lineage; Fig. 4). This is in stark contrast to the situation in mitochondria, which exhibit a plethora of different non-canonical codes, including those with stop-to-sense reassignments of UAG (encoding leucine independently in some chytrids and green algae; see above) or UAA (encoding tyrosine in some flatworms [50]), but no known case of a simultaneous reassignment of both UAG and UAA. There might be some inherent molecular predisposition for different evolutionary trajectories of the UAG and UAA codons in nuclear and mitochondrial codes (for example, related to the differences in the mechanism of translation termination), but the discovery of the two new code variants in the rhizarian exLh and *I. spirale* finally indicates that this principle is not absolute. Our study thus provides an important new perspective on the evolution of the genetic code in eukaryotes. In addition, we developed a new generally applicable phylogeny-informed method for inferring the meaning of reassigned codons that will facilitate characterization of non-standard genetic codes to be discovered in the future.

Our research has also contributed to improving our knowledge of the phylogenetic diversity of eukaryotes. The discovery of a new insect-associated lineage of Rhizaria, interesting in itself, may be of a special significance, because the host species, the heteropteran *L. hesperus*, is an important pest of several crop plants, especially cotton [25, 51]. The second subject of our study, *I. spirale*, is also a biologically interesting organism from an underexplored area of the eukaryote phylogeny. No genome-level sequence data has been reported so far from non-diplomonad fornicates, so our sequencing of the *I. spirale* transcriptome fills in an important gap in the sampling of eukaryotic diversity and will enable us to investigate other aspects of the biology of *I. spirale* (and fornicates in general).

## Methods

### Assembling the 18S rRNA sequence of the rhizarian exLh from RNA-seq data

Searching the TSA from *L. hesperus* [25, 52] with blastn and the previously reported *L. hesperus* 18S rRNA gene sequence (U06476.1) as a query identified five contigs as significantly similar hits. Three of them (GBHO01018850.1, GDHC01017703.1, and GDHC01007684.1) were identical or nearly identical in the region aligned with the query, so they apparently come from the insect itself and were not investigated further. The remaining two contigs, GBHO01018845.1 and GDHC01015840.1, included a partial 18S rRNA-related region of a different length, but identical between the two contigs along a segment of ~500 bp, corresponding to the 3’-end of the 18S rRNA molecule. This partial 18S rRNA sequence did not closely match any sequence in the GenBank database and was very different from the 18S rRNA sequences of *L. hesperus* (or other insects) and its symbiotic trypanosomatid *Blastocrithidia* sp. [18], suggesting that it comes from a third, unknown organism. We hypothesized that it is derived from the same organism as the Rhizaria-related component detected among protein-coding transcripts. Careful examination of these two contigs by blast searches against the NCBI sequence databases and against the raw Illumina reads from *L. hesperus* in the NCBI Sequence Read Archive (SRA) database indicated that both are in fact chimeric and resulted from an artificial merging of separate transcripts, as described in detail in the following two paragraphs.

The contig GBHO01018845.1 comes from the first *L. hesperus* TSA version [25] and proved to consist of a segment representing the 18S rRNA 3’-end region of the unknown organism (positions 1–862) fused to a segment (the rest of the contig) corresponding to the ITS1-5.8S–ITS2-28S rRNA region of an rRNA locus most similar the respective sequences of close relatives of *L. hesperus* (heteropterans of the Miridae family). The latter was thus interpreted by us as the actual rRNA locus of *L. hesperus* (which has not been reported before and is not represented in the NCBI nr nucleotide database). Examination of Illumina reads mapped onto this contig and detailed sequence comparisons revealed that the artificial fusion of the two segments was due to the fact that the 18S rRNA from *L. hesperus* and the 18S rRNA of an unknown origin share a block of 33 identical nucleotides that corresponds to a highly conserved region of the 18S rRNA molecule close to its very 3’-end. Iterative searches of the raw Illumina RNA-seq reads from *L. hesperus* enabled us to unambiguously extend the 5’-end of the enigmatic sequence by more than 1000 bp to cover the full length of a typical eukaryotic 18S rRNA sequence. This newly assembled part is for unknown reasons completely missing from the *L. hesperus* TSA. Nevertheless, we did not notice any ambiguities in the alignment of RNA-seq reads along the sequence and a large part of the reassembled sequence was confirmed by PCR and sequencing (see the next section), corroborating our interpretation of the RNA-seq data.

The second contig under scrutiny, GDHC01015840.1, comes from the newer *L. hesperu*s TSA version [52], which was released only after our analyses of protein-coding transcripts (related to the new genetic code in the rhizarian exLh had been completed). It proved to be likewise an artefact. The first part (from the 3’-end to the position 656 in the reverse complement orientation) corresponds to an mRNA (including a putative long 3’ untranslated region without any similar hits) that encodes a predicted protein kinase. The best database hits of this kinase come from trypanosomatids and exhibit the same unusual genetic code as described for the *L. hesperus*-residing *Blastocrithidia* sp., so this sequence is simply one of the many *Blastocrithidia* sp.-derived sequences identified in the *L. hesperus* TSA by Záhonová et al. [18]. The region 655–136 (again in the reverse complement orientation) of the contig GDHC01015840.1 is identical to a region of the 18S rRNA sequence reassembled on the basis of the contig GBHO01018845.1. The third part (positions 136–1) is identical to the 3’-end of 18S rRNA sequences from various lepidopterans, indicating another source of contamination in the more recently reported *L. hesperus* TSA data [52]. We did not investigate in detail what might be the factors that misled the program used for the RNA-seq reads assembly to create the tripartite chimera (i.e., the contig GDHC01015840.1), but some short regions of sequence identity between different RNA molecules or chimeric reads are the likely explanation.

The reassembled full-length 18S rRNA sequence, attributed by a phylogenetic analysis (Fig. 1a) to the rhizarian exLh, is provided in Additional file 7.

### PCR-based detection of the rhizarian exLh in *L. hesperus*

Total DNA from five specimens of *L. hesperus* collected in September 2015 (Kern County, CA) and provided by Dr. Surendra K. Dara (University of California Cooperative Extension) was extracted using the NucleoSpin^®^ Tissue Kit (Macherey-Nagel, Germany) according to the manufacturer’s instructions. Two overlapping 18S rRNA gene fragments were amplified by PCR using two sets of primers designed on the basis of the 18S rRNA sequence of the rhizarian exLh assembled from RNA-seq reads (see above): Rhiz-N-F1 (5’-AGCGAAAGCATTCACCAAGT-3’) and Rhiz-N-R1 (5’-TCCTTCCTTCGGCTAGAACA-3’); Rhiz-N-F2 (5’-CCTAAATAACTCTCTGCCCTATC-3’) and Rhiz-N-R2 (5’-ACCTGTCTATCCTCACTATATCC-3’). The reaction was performed in 50 μL of a cocktail containing 3 mM MgCl_2_, 1 mM dNTPs, both in MyTaq™ Reaction Buffer Red (Bioline, UK), 0.025 U/μL MyTaq™ DNA polymerase (Bioline, UK), and 0.4 μM each primer, with an annealing temperature of 57 °C (Rhiz-NF1/R1 primers) or 48 °C (Rhiz-NF2/R2 primers). PCR products were purified using GeneJET PCR purification kit (Thermo Scientific, USA) and directly sequenced with the Sanger dideoxynucleotide method using the amplification primers. Sequencing reads were edited and assembled using Sequencher v5.0 program. The final sequence was deposited at GenBank with the accession number KY201455.

### Sequencing and assembly of the *I. spirale* transcriptome

*I. spirale* cells (strain PHEM1, monoeukaryotic xenic culture) were cultured at room temperature for 7 days before RNA extraction (see [30] for details on the culturing). Cells from 50 mL of the culture were pelleted by double centrifugation (1200 *g* for 8 min and 2000 *g* for 12 min). Total RNA was extracted using TriReagent Solution (Ambion, USA) according the manufacturer’s instructions and mRNA was isolated by double polyA selection with Dynabeads Oligo(dT)25 (Invitrogen, USA). Illumina RNAseq libraries were prepared from mRNA using the NEXTflex RNA-Seq Kit (developed by BIOO Scientific) and 150 bp paired end reads were sequenced on the MiSeq platform and quality-filtered by the Genomics Core Facility, EMBL, Heidelberg. A total of 14,016,257 read pairs were further processed by removing adapters using Cutadapt v1.3 [53] and assembled into contigs using the Trinity package v2.0.6 [54], resulting in 38,331 contigs (Trinity transcripts), 29,865 of which were longer than 250 bp. Possible contamination of the transcriptome assembly by another eukaryote was tested by identifying homologs of cytoplasmic ribosomal proteins (generally encoded by highly expressed genes, hence suitable markers for possible low-abundance contamination) and inferring their phylogenetic position with respect to orthologs from a reference set of phylogenetically diverse eukaryotes (the phylogenetic analysis is described below). No obvious eukaryotic contamination was detected by this approach (Additional file 3: Table S2A). However, the transcriptome assembly was heavily contaminated by bacterial sequences (derived from bacteria coinhabiting the *I. spirale* culture). Hence, non-eukaryotic contigs, defined as those with the five best blastx hits of the archaeal, eubacterial, or viral origin in the trEMBL database (release 2016_10), were removed prior to deposition of the assembly to the TSA database.

### Read mapping

Reads from the RNA-seq projects for *I. spirale* (our new data) and *L. hesperus* [25] (SRR1186295, SRR1186315, SRR1186516, SRR1186658, SRR1186749, SRR1186752, SRR1186787, SRR1186788, SRR1186789) were indexed, quality trimmed using Trimmomatic v0.32. [55], and aligned on the assembled transcripts using Bowtie2 v2.1.0 in a fast local mode [56]. The output file was then converted into a binary format (BAM), sorted and indexed using Samtools v0.1.19 [57]. Positions of special interest were checked by visual inspection of mapped reads using Tablet v1.15.09.01 [58] and Integrative Genomic Viewer v2.3.68 [59]. All output files are available upon request from the corresponding author.

### Sequence searches and alignments

The publicly available TSA from *L. hesperus* (its first version reported in [25]) and the newly obtained TSA from *I. spirale* were searched by tblastn to identify protein-coding transcripts of interest for subsequent phylogenetic and genetic code analyses. As queries we used protein sequences from sets of orthologs employed in previously published phylogenomic studies [60, 61] or from our in-house broad collection of sequence alignments. Significantly similar hits representing likely orthologs of query sequences were subjected to conceptual translation employing the standard genetic code to obtain working versions of encoded protein sequences (in-frame UAG codons were allowed and translated as an unknown amino acid – X). The deduced protein sequences were aligned with their putative orthologs and ML trees were inferred for each alignment to detect possible paralogs or contamination from non-target species. Nucleotide and deduced amino acid sequences of detected orthologs and the trees are available in Additional file 1: Table S1A–C). Given the fact the 71 sequences attributed to the rhizarian exLh come from a transcriptome assembly representing a mixture of three eukaryotic organisms (the insect *L. hesperus*, the trypanosomatid *Blastocrithidia* sp., and the rhizarian), we checked them all for possible chimerism by careful inspection of raw sequencing reads mapped onto the assembled contigs. No signatures of artefacts were noted, except for three contigs (GBHO01012272, GBHO01017741, GBHO01017234) that each proved to include a region that we attributed to the rhizarian exLh, linked to a region apparently representing a gene from the insect. These chimeras resulted from misassembly due to local sequence similarities in non-coding regions without affecting the authenticity of the coding sequences. As an additional test of the correct assignment of the analyzed sequences to the rhizarian exLh we more closely checked the eight transcripts (of the 71 mentioned above) that returned at least one metazoan or trypanosomatid hit among the best 10 blastx hits. We divided the respective predicted protein sequences into two halves and computed a phylogenetic tree for each segment independently. None of these segments branched specifically with metazoan or trypanosomatid sequences, indicating that the eight sequences tested are unlikely to be chimeric.

The analysis of 18S rDNA sequences aimed at defining the position of the rhizarian exLh (Fig. 1a) was based on a combination of the published datasets [27, 62]. The multi-locus phylogenetic analysis of the Fornicata (Fig. 1b) was based on the dataset published by Takishita et al. [32] expanded by the addition of the respective orthologs from *I. spirale* (including a complete 18S rRNA sequence identified in the transcriptome, provided in Additional file 7). For the final analysis we did not include two markers – alpha- and beta-tubulin, because their *I. spirale* orthologs proved to be extremely divergent. All alignments were built using MAFFT 7.245 with the G-INS-i algorithm [63] and manually trimmed to exclude unreliably aligned regions. The final 18S rDNA sequence alignment including the rhizarian exLh sequence comprised 1491 positions. Two multi-locus datasets were created by concatenating the relevant individual alignments using SequenceMatrix v1.8 [64]. The final 70-protein eukaryote-wide phylogenomic matrix including 54 orthologs from the rhizarian exLh (listed in Additional file 1: Table S1A–C) and 60 orthologs from *I. spirale* (listed in Additional file 1: Table S1A, B) comprised 21,145 aligned amino acid residues. The final five-locus matrix for the analysis of the fornicate phylogeny comprised 3589 aligned characters (1229 nucleotides of 18S rRNA and 2360 amino acid residues coming from sequences of HSP70, HSP90, EF1-α, and EF2 proteins).

For the sequence analysis of the eukaryotic release factor 1 (eRF1), we combined the dataset published by Swart et al. [16] with selected homologs from the GenBank and MMETSP databases. We also added the eRF1 sequences from both species investigated in this study (the rhizarian exLh and *I. spirale*) and from *Trentepohlia iolithus* (a representative of ulvophyte algae with a non-canonical genetic code) that we detected in our assembly of publicly available transcriptome reads from this species (SRX387803). Accession numbers of the analyzed eRF1 sequences are provided in Additional file 6: Figure S2.

### Phylogenetic and phylogenomic analyses

Most ML phylogenetic analyses (except the analysis using IQ-TREE, see below) were done using RAxML v8.2.8 [65] and run at the CIPRES Science Gateway [66] or the National Grid Infrastructure MetaCentrum. Single-protein ML trees were inferred with RAxML using the PROTGAMMALG4X substitution model with 100 rapid bootstrap replicates. The 18S rRNA gene tree was constructed using the GTRGAMMAI model and 500 rapid bootstraps. The phylogenomic analysis of the concatenated alignments of 70 conserved proteins was constructed with RAxML using the PROTGAMMALG4X model; branch support was estimated from 500 rapid bootstrap replicates. Because RAxML is not equipped with site-heterogeneous models, we also ran an analysis of the same dataset with IQ-TREE v. 1.3.8 [67] using the LG + Γ4 + C20 model with class weights optimized from the dataset and the exchangeabilities from the LG Q-Matrix (LG + Γ4 + FMIX{empirical,C20pi1…C20pi20}) [68]. Branch support was estimated from 1000 ultrafast bootstrap replicates. The dataset consisting of concatenated alignments of 18S rDNA and four proteins was analyzed with RAxML using the GTRGAMMA (for the 18S rDNA partition) and the PROTGAMMALG4X (for the protein sequence partition) model and 500 rapid bootstraps. For this and the global eukaryotic 18S rDNA data set, we also used Bayesian analyses employing site-heterogeneous substitution models. We ran PhyloBayes MPI v1.5a [69] using the CAT-Poisson + Γ4 model (five-locus data set) and the CAT-GTR + Γ4 model (18S rDNA data set). Two independent chains were run until they converged (i.e., their highest observed discrepancy was lower than 0.1 and the effective sample size of all model characteristics was at least 100). Consensus trees and posterior probabilities were calculated by sampling every 50th tree, with the first 25% of the generations discarded as burn-in.

### Codon analyses

Several approaches were used to define the amino acid residue encoded by the in-frame UAG codons in transcripts from the rhizarian exLh and *I. spirale*. In the first method, we used eukaryote-wide sequence alignments of orthologous proteins to identify hyperconserved positions that correspond to an in-frame UAG codon in the underlying coding sequence from the rhizarian exLh or *I. spirale*. Specifically, we visually inspected the alignments to identify well-aligned blocks that include at least one UAG site (defined as UAG positions flanked by well conserved blocks of five amino acids on both sides). For the rhizarian exLh we based the analysis on the 54 alignments of orthologs used previously in the phylogenomic analysis (see the previous section), which included 192 UAG-encoded amino acid positions. For *I. spirale* we started with a broad in-house set of taxonomically rich alignments of 431 orthologous proteins, which were simplified by keeping only sequences from 54 reference species. Further analysis was restricted to those alignments that included sequences from both *I. spirale* and its specific relative, the diplomonad *Giardia intestinalis*; 64 of them contained at least one UAG-encoded amino acid in a conserved block, in total representing 96 cases (some proteins had more than one such position). The hyperconserved UAG-containing positions were defined as those with a particular amino acid residue conserved in at least 90% of the sequences in the alignment. This threshold was passed by 86 out the 192 UAG-containing positions in the rhizarian exLh and 28 out of the 96 positions in *I. spirale*. The results were plotted as graphs using RStudio v0.99.491 [70].

In the second analysis, we estimated the most likely amino acid encoded by the UAG codon using a newly devised phylogeny-informed ML-based method. All ML calculations described below were done using IQ-TREE and the LG + Γ substitution model. We first constructed a reference tree separately for the analyses aimed at the rhizarian exLh and *I. spirale*. The reference tree for the former organism was inferred using a concatenation of alignments of eight conserved proteins (Ap1m1, Atp6v1a, Bat1, Dkc1, Drg2, Eef2, Eif5b, and Etf1; Additional file 1: Table S1*C*) containing in total 71 positions with in-frame UAG codons in the rhizarian exLh sequences. The reference tree for the *I. spirale*-specific analysis was inferred from a concatenation of the well-aligned blocks of 11 amino acid residues (together including 96 in-frame UAG codons in *I. spirale* sequences) that were also used in the aforementioned analysis based on hyperconserved positions. In the alignments used for the calculation of the reference trees, in-frame UAG codons were represented as an undetermined amino acid (X). Subsequent probability calculations were conditional upon, or assuming that, UAG codes for some single particular amino acids at all positions of the alignment, i.e., we were comparing 20 possible reassignments. Consequently, we prepared 20 modifications of each concatenated alignment, each with one of the possible amino acids considered at all positions corresponding to the in-frame UAG codons. A ML tree was then inferred for each of the 20 datasets, using the LG + Γ model with the alpha parameter fixed to the value estimated for the concatenated alignment with UAG encoded as X and employing the reference species tree with fixed branch lengths as a constraint. The resulting trees were ordered according to the log-likelihood score (Table 1). The dataset giving the highest score predicts the most likely amino acid encoded by the UAG codon in the respective species. To test the statistical significance of this assignment, we calculated for each of the 20 possible amino acids the conditional probability that UAG codes for the amino acid using the likelihood scores computed before. The conditional probability that the organism translates UAG codons as a given hypothesized amino acid is equal to the ratio of the likelihood score of the alignment with this amino acid at UAG positions to the sum of the likelihood scores for 20 alignments, each of which decodes UAG by a particular amino acid. The conditional probabilities for each amino acid (Table 1) give overwhelming support for the hypothesis that UAG encodes leucine in rhizarian exLh and glutamine in *I. spirale*.

Given the scarcity of UAG codons in *I. spirale* sequences, we carried out a more systematic analysis of the transcriptome. To exclude sequences of bacterial origin and transcripts that may contain frame-shift errors or retained introns, we used blastx to search against the trEMBL database (release 2016_10) and identified contigs that met the following two criteria: all five best hits are of eukaryotic origin, and all high-scoring segment pairs (hsps) with the best hit correspond to the same reading frame in the contig. These conditions retrieved 3064 contigs, which were conceptually translated in the reading frame corresponding to the blastx match with UAG decoded as X (i.e., an undetermined amino acid). The resulting amino acid sequences were searched with blastp against the trEMBL database and filtered by keeping only those that returned at least 50 hits with e-value < 1 × 10^–30^ to ensure that a sufficient number of close homologs is available for comparison with the *I. spirale* sequence. In addition, only a single representative sequence was kept (the one that gave blastp hits with the lowest e-value) for groups of sequences corresponding to alternative transcripts of the same gene. This filtering retained 730 sequences (Additional file 3: Table S2*D*), each of which was aligned with the 50 best blastp hits using MAFFT. The alignments were inspected using a custom Python script for cases where the *I. spirale* sequence included at least one position corresponding to the UAG codon clearly within the coding sequence of the underlying transcript (i.e., not in regions likely corresponding to 5’- or 3’-UTRs). For further analyses, we considered only conserved positions, defined using a more stringent (90%) or a more permissive (50%) threshold for amino acid identity across the alignment (Fig. 2e). As a control, we did the same analysis for positions corresponding to canonical glutamine codons in *I. spirale* sequences (Fig. 2f). We additionally inspected the 730 alignments defined above for the presence of UAA or UAG codons that would interrupt the coding sequence of the *I. spirale* transcripts, but we did not find any convincing cases (see Results and Discussion).

To test the possibility that UAG may simultaneously serve as a *bona fide* termination codon in *I. spirale* transcripts, we checked the 730 *I. spirale* sequences defined above to identify for each *I. spirale* sequence the most likely termination codon. Specifically, to simplify the analysis we selected those sequences that gave a single hsp with the best hit in the initial blastp search (keeping 574 sequences) and check the first in-frame termination codon (UAG, UAA, UGA) downstream of the respective hsp. The cases where the first such codon was UAG were checked manually by inspecting the multiple alignment with homologous sequences. In all 81 *I. spirale* sequences of this category, the respective UAG was found to more likely correspond to a sense codon (presumably encoding glutamine) further downstream followed by a true termination codon represented by UAA or UGA.

Relative codon frequencies (Fig. 3) were computed from identified coding regions in two selected subsets of transcripts from the rhizarian exLh (Additional file 1: Tables S1A and S1B) and for the full reference set of 126 transcripts from *I. spirale* (Additional file 3: Table S2A) as the count of a specific codon divided by the total number of codons encoding the same amino acid [29].

## Additional files

Additional file 1: Table S1. List of detected genes, single-gene trees, and hyperconserved regions for the rhizarian exLh. (XLSX 682 kb)
Additional file 2: Figure S1. Phylogenomic analysis of eukaryotes including the rhizarian exLh and *I. spirale* based on 70 conserved proteins. (PDF 603 kb)
Additional file 3: Table S2. List of analyzed transcripts, single-gene trees, and hyperconserved regions for *Iotanema spirale*. (XLSX 787 kb)
Additional file 4: Table S3. Stop codons in available transcripts of *Guttulinopsis vulgaris*. (XLSX 112 kb)
Additional file 5: Table S4. tRNA genes and the number of their paralogs in selected eukaryotic genomes. (XLSX 15 kb)
Additional file 6: Figure S2. Sequence comparison of the conserved N-terminal domain of the eukaryotic release factor 1 (eRF1) from various eukaryotes with different types of the nuclear genetic code. (PDF 150 kb)
Additional file 7: Complete 18S rRNA sequences of rhizarian exLh and *Iotanema spirale* (re)assembled from RNAseq reads. (TXT 7 kb)
