# Supplementary material for: Nuclear genetic codes with a different meaning of the UAG and the UAA codon
Source: BMC Biol. 2017 Feb 13;15:8. doi: 10.1186/s12915-017-0353-y (PMC5304391; doi:10.1186/s12915-017-0353-y)
Supplement: Additional file 2: Figure S1. — Phylogenomic analysis of eukaryotes including the rhizarian exLh and I. spirale based on 70 conserved proteins. (PDF 603 kb) [file 12915_2017_353_MOESM2_ESM.pdf]

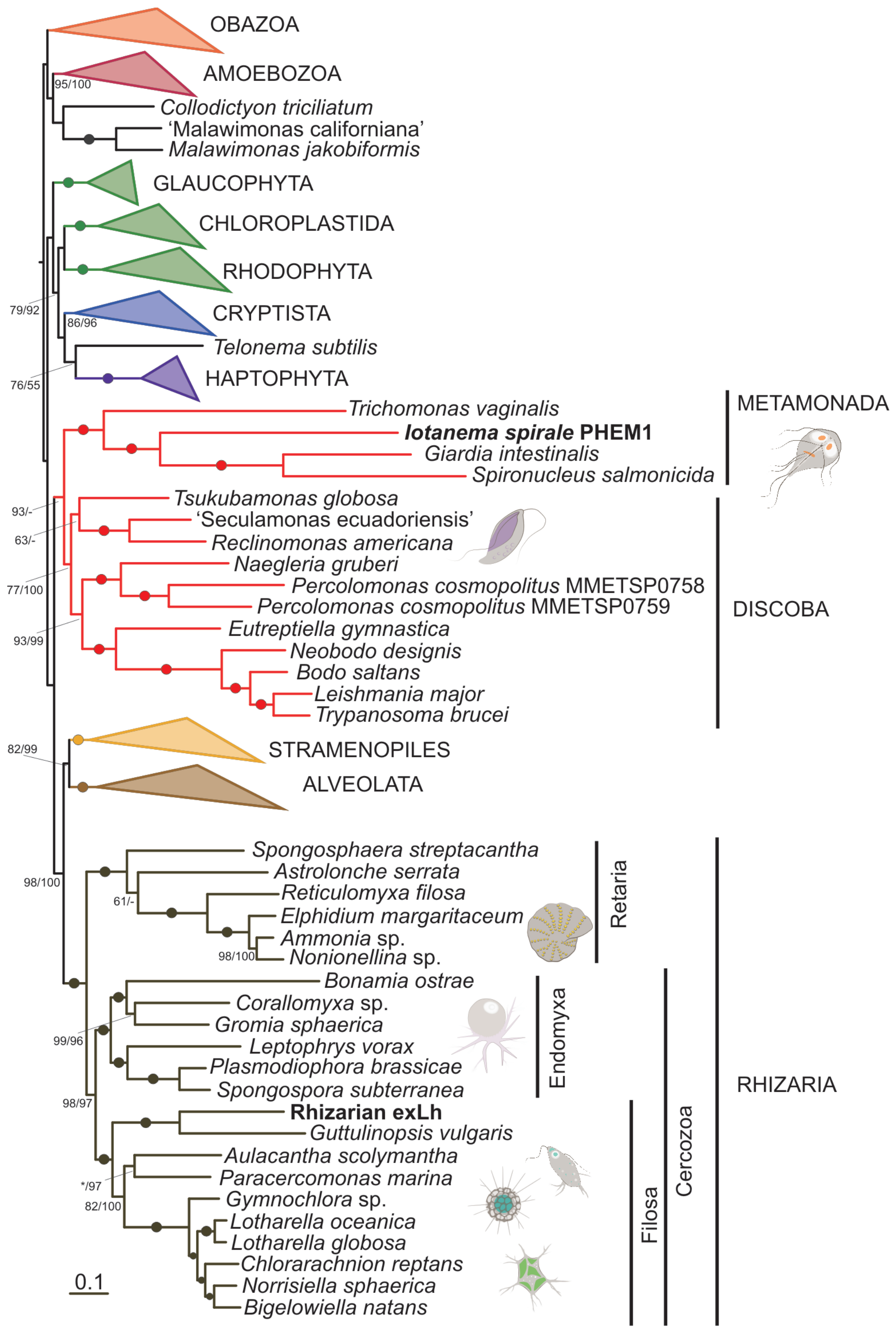

Fig. S1. Phylogenomic analysis of eukaryotes including the rhizarian exLh and *I. spirale* based on 70 conserved proteins. The ML tree was constructed with RAxML 8.2.8 using the PROTGAMMALG4X model. The values at branches represent RAxML BS values estimated from 500 rapid bootstraps followed by IQ-tree BS values estimated from 1000 ultrafast bootstrap replicates (LG+Γ4+C20 model with class weights optimized from the dataset). Black dots indicate maximal statistical support. Asterisks indicate support values lower than 50% or 0.5, dashes mark branches in the ML tree that are absent from the PhyloBayes tree.
