# Supplementary material for: Nuclear genetic codes with a different meaning of the UAG and the UAA codon
Source: BMC Biol. 2017 Feb 13;15:8. doi: 10.1186/s12915-017-0353-y (PMC5304391; doi:10.1186/s12915-017-0353-y)
Supplement: Additional file 6: Figure S2. — Sequence comparison of the conserved N-terminal domain of the eukaryotic release factor 1 (eRF1) from various eukaryotes with different types of the nuclear genetic code. (PDF 150 kb) [file 12915_2017_353_MOESM6_ESM.pdf]

```
                                    30        40        50        60        70            130
                                    .|....|....|....|....|....|....|....|....|..     .|....|..
Homo sapiens ***                    GNGTSMISLIIPPKDQISRVAKMLADEFGTASNIKSRVNRLSVL // LYLCDNKFH
Arabidopsis thaliana ***            ..........M..R..V...T...G..Y............Q... // .........
Dictyostelium_discoideum ***        ...........R.G...AK.N....E.Y................ // .........
Thecamonas trahens ***              S......T..A..G..V.SLTRL.T..A.S.............. // M.M..SR..
Emiliania huxleyi ***               ..........L..GS...QAS....E.Y................ // .........
Guttulinopsis vulgaris ***          .....V...V....T.L.Q.SQ..IT.E............Q... // ..M...R..
Giardia intestinalis ***            .S...V...L...GE.L.S.TG..TN.Y...........KNA.. // I....S...
Roombia truncata ***                .............R.....................N........ // .........
Trichomonas vaginalis ***           .D....VTIL...G.AVH.MRQK.T..L.......N....Q..E // ..I..RR..
Reticulomyxa filosa ***             .........VV.........Q...Q..H....H........... // .........
Entamoeba histolytica ***           .D.....T..MK.ME...KMMQK.VE.Y...T........Q... // T.....R..
Nannochloropsis gaditana ***        .............R......N......Y...T.....T...... // .........
Ostreococcus lucimarinus ***        .........V....G.VNL.Q...IE.M...T........Q... // ..M......
Reclinomonas americana ***          ..........L..GE...AN....SE.Q...T........Q... // ......R..
Telonema subtilis ***               ..........L....D.A.A....G......Q............ // .........
Symbiodinium minutum ***            .....A.T..LNA.QD.NLTN...TE.Y....C........... // M....S...
Didinium nasutum ***                .S.....T...NF....NIH.R...E.V.K........T.QN.T // ..K..SV..
Dileptus margaritifer ***           .S.........NY....NPFM...V..V.K........T.QN.T // ..R..SV..
Litonotus pictus ***                .S.....T...NS.....PSMR..TE.V.K.T......T.QN.T // ..R..SV..
Climacostomum virens ***            .....L......SR..LNFAS.LVTE.Y.K.A......V.QA.Q // .........
Protocruzia adhaerens ***           ............A...VAKAN...VE..........TT..Q... // ......R..
Fabrea salina ***                   .....L..........LPMTN..ITE.Y.K.A......V.NA.Q // ..I.....Q
Blastocl. emersonii ***             ..........L...E.LP..N......Y.A.............. // M.M......
Naegleria gruberi ***               .................A..T...QE.Y............I..Q // .........
Trypanosoma brucei ***              .M...V..IYMT..E...GMVTK.NN.Y........HT.K...Q // .........
Euglena gracilis ***                .......T.MLT.....ALMT.K.NE.Y...T....H..K.... // M.M......
Holosticha sp. QQ*                  ......V..VV....D.N..S.L..TM.LS..Q.....QT.Q..I // ..F.GG..Q
Tetrahymena thermophila QQ*         .S....V.......K..NDST.LIS...SK.T...D....Q..Q // ..F..S...
Sterk. histriomuscorum QQ*          ......V........D.NKYG.L.TG.MSA.Q.....ITKQ..V // ..F.GG..Q
Paramecium tetraurelia QQ*          TA...VV.VY....RI..DITNR.NTQYAE.AS..DKG..I..Q // ..F..PQ..
Loxodes striatus QQ*                .....L........E...LINQK.T.AH.R.Q....KAVQKA.Q // FF..E.T.Y
Gonostomum sp. QQ*                  ......V..V.....DVM..N.L.TQ.ISS.G....SKT.Q..Q // ..F...S.K
Pseudoco. persalinus QQ*            STT......L...NK...DYT...T..ISQ.NS..D....QA.Q // I.R..S..Y
Eschaneustyla sp. QQ*               .......T..V....D.NIIT.KVTQ.LSS.Q.....TT.Q..I // A.Y...R..
Urostyla sp. QQ*                    ......VT.VV...ED.NM.S.K.TQ.LSS.QS...KTT.S... // ..F...S..
Oxytricha trifallax QQ*             ......V........D.NKYG.L.TG.MSA.Q.....ITKQ..V // ..F.GG..Q
Uroleptus sp. QQ*                   ......V..V.....D.N.SG.L..S.LSA.N.....IT.Q... // M.F.GG..Q
Paraurostyla weissei QQ*            ......V........D.N.SG.L.VQ.LSA.N.....IT.Q... // M.F.GG..Q
Stichotrichida sp. QQ*              ......V...V....DVN..G.L.TQ.LSQ.Q.....ITKQ..I // ..F.GG..Q
Tetmemena pustulata QQ*             ......V...V....D.NKYN.LIVG.LSA.Q....KITKQ..T // ..F.GG..Q
Ichthyop. multifiliis QQ*           .Q....V...L...K...DAT.L.NE.Y.K.T...D.I..Q..Q // FIFVIVN.M
Strombidium inclinatum QQ*          ......VT.V....EAVAQ.TSF.NK.YAE.A....KQTMT..Q // M.K.E.R..
Pseudokerenopsis sp. QQ*            .......T.VV....D.NIIT.K.NL.QSS.Q...N.QT.Q..M // V.F...S..
Flavella ehrenbergii QQ*            .....FV..Y..AG.PVTKHMQL..S.LAG.ES....QT.Q... // A.K.Q.T.Y
Aristerostoma sp. QQ*               ......V..V....K.L.DITQK.TE.I.A......NIV.K... // Q....SS..
Uronema sp. QQ*                     CS.....T.V...NK.MAEFTRH.TT.YSE.NS..D.A..Q..Q // F.R..S..Y
Spironucleus salmonicida QQ*        T.S..VV..VM..GED.NKMVQ..NQ.ATQ.DC....Q..QA.Q // M.S..GV..
Trentepohlia iolithus QQ*           ...-.....YM....VAK//VS..TE.Y.....V......N.I. // ..R.E...I
Carchesium polypinum EE*            IS.-.VVTI.M..RKNVAEIT......Q.K.AS..DKT.KNA.I // I.Y..S..Y
Mesodinium pulex YY*                IQ.-.C..I.....A...DTVTK.TE.Y.V......KLT.T... // I.F..G...
Iotanema spirale *Q*                C..G.L.TA...AG...TMMSHR.VT.Y.A.............. // I.H.GGQ.V
Rhizarian exLh  *L*                 ......V..VV.....VP.LCS.-TN.IA..V.....I..A..I // ..R...R..
Blepharisma americanum **W          .....L.......RE.LPIIN..ITE.Y.KS......IV.QA.Q // ..I......
Blepharisma japonicum **W           .....L.......RE.LPIIN..ITE.Y.KS......IV.QA.Q // ..I......
Euplotes focardii **C               .........LLS.R.A..K.QG...G.M...T.......KQA.. // K.I...R.C
Euplotes crassus **C                .........LLS.R.A..K.QG...T.M...T........QA.. // K.I.....C
Eupl. octocarinatus/B **C           .D.......LLS.R....K.QA...G.A...V........QA.. // K.I.....C
Eupl. aediculatus/B **C             .........LLS.R....K.QG...G.A...V.........A.. // K.I.....C
Eupl. octocarinatus/A **C           .........LLS.R.A.PK.QG...G.Y...ES...KI...A.Q // K.I.....Y
Eupl. aediculatus/A **C             ....G....LLS.R.A..K.QG..SS.S...ES.......QA.T // K.I.....Y
Nyctotherus ovalis **-              .............GE..TKMQ.K.I..G.K..........Q..E // ..Y.GER.D
Nyctotherus ovalis/B **-            .............GE..AKMQ.K.I..G.K..........Q..E // ..Y.GER.D
Parduczia sp. QQ(W)                 .H...L.T..........LWN.NINE...K.AQ....LV.Q..Q // M....SR.N
Condylostoma magnum (QQW)           .....L.......RE..NLIN.QMN..L.K.A......V.GA.Q // ..M..SR..
Blastocrithidia sp. (EE)W           .M...V..AY.T..E.VAS.VTK.NN.Y........HS.KIG.Q // M........
```

Fig. S2. Sequence comparison of the conserved N-terminal domain of the eukaryotic release factor 1 (eRF1) from various eukaryotes with different types of the nuclear genetic code. Only two parts of the alignment containing motifs that are thought to be important for stop codon recognition are shown (separated with a double slash). The type of the genetic code is indicated to the right of the species name by indicating the meaning of UAA, UAG, and UGA codons (in this order) as follows: asterisk – termination codon, one-letter amino acid abbreviation – sense codon with the respective specificity. Codons with context-dependent dual meaning (sense when in-frame, stop when at the end of the coding sequence) are

indicated with the respective amino acid abbreviation in brackets. The human eRF1 sequence (on the top) is used as a reference (with the numbers above indicating the actual position within the full-length sequence), identity with the reference is indicated as a dot in the other sequences. Functionally significant motifs and positions are printed in bold in the reference sequence and the respective regions are highlighted with a grey background throughout the alignment. Amino acid residues in these regions deviating from the canonical sequence are highlighted with a light blue background.

Accession numbers of used eRF1 gene sequences (GenBank or MMETSP database): *Homo sapiens* NP_004721, *Arabidopsis_thaliana* AT1G12920.1, *Dictyostelium_discoideum* EAL63131.1, *Thecamonas trahens* XP_013755399.1, *Emiliania huxleyi* XP_005786825.1, *Guttulinopsis vulgaris* JT844977.1, *Giardia intestinalis* ESU42869.1, *Roombia truncata* JP445973, *Trichomonas vaginalis* XP_001317480.1, *Reticulomyxa filosa* ETO12436.1; *Entamoeba histolytica* XP_655687.1, *Nannochloropsis gaditana* EWM25096.1, *Ostreococcus lucimarinus* XP_001417750.1, *Reclinomonas americana* EC793298.1, *Telonema subtilis* FN392508.1, *Symbiodinium minutum* BASF01007484.1, *Didinium nasutum* AB086368.1, *Dileptus margaritifer* AB086369, *Litonotus pictus* CAMNT_0010815823, *Climacostomum virens* CAMNT_0052066353, *Protocruzia adhaerens* CAMNT_0002413411 (MMETSP), *Fabrea salina* CAMNT_0049035673 (MMETSP), *Blastocladiella emersonii* CO974532.1, *Naegleria gruberi* XP_002682100.1, *Trypanosoma brucei gambiense* DAL972 XP_011779402, *Euglena gracilis* EC680368.1, *Holosticha* sp. AAT39329.1, *Tetrahymena thermophila* XP_001018735, *Sterkiella histriomuscorum* Q9BMX3.1, *Paramecium tetraurelia* XP_001425245, *Loxodes striatus* Q5CD84.1, *Gonostomum* sp. AAT39330.1, *Pseudocohnilembus persalinus* KRX05899.1, *Eschaneustyla* sp. AAT39331.1, *Urostyla* sp. AAT39328.1, *Oxytricha trifallax* AAK12090.1, Uroleptus sp. AAT39327.1, *Paraurostyla weissei* AAT39326.1, Stichotrichida sp. Alaska AAN62563.1, *Tetmemena pustulata* AAN62568.1, *Ichthyophthirius multifiliis* XP_004032541, *Strombidium inclinatum* CAMNT_0010754279 (MMETSP), *Pseudokerenopsis* sp. CAMNT_0010847507 (MMETSP), *Flavella ehrenbergii* CAMNT_0010732437, *Aristerostoma* sp. CAMNT_0001789297, *Uronema* sp. CAMNT_0038737683 (MMETSP), *Spironucleus salmonicida* EST45466.1, *Trentepohlia iolithus* (newly assembled contig, SRR1044982), *Carchesium polypinum* (c22299_g1_i1, SRX849478), *Mesodinium pulex* CAMNT_0004595873, *Iotanema spirale* a12404c01, Rhizarian exLh GBHO01004851, *Blepharisma americanum* AF317831.1, *Blepharisma japonicum* CAC16186.1, *Euplotes focardii* CAMNT_0053081483.1, *Euplotes crassus* (MMETSP), *Euplotes octocarinatus*/B AAG25924.1, *Euplotes aediculatus*/B AAK07830.1, *Euplotes octocarinatus*/A CAC14170, *Euplotes aediculatus*/A AAK07829.1, *Nyctotherus ovalis* AAX19092.1, *Nyctotherus ovalis*/B AAX19093.1, *Parduczia* sp. CAMNT_0047593165 (MMETSP), *Condylostoma magnum* CAMNT_0008277929 (MMETSP), *Blastocrithidia* sp. GDHC01021218.1.
